# S7-1 Physical activity and planetary health

**DOI:** 10.1093/eurpub/ckad133.033

**Published:** 2023-09-11

**Authors:** Chair(s):, Karim Abu-Omar

**Affiliations:** WHO Collaborating Centre for Physical Activity and Public Health, Friedrich-Alexander Universität Erlangen-Nürnberg

## Abstract

The symposium intends to showcase research that seeks to connect physical activity to planetary health. Such research is warranted by the thread of global warming and the loss of biodiversity, but at the same time it is still in its early stages and many open questions remain. Concepts such as one health and planetary health have now been developed and provide more general frameworks on how to map the impacts of (health) behavior on the environment. However, while these frameworks are still quite new for our field of study, it is worthwhile exploring how they can be utilized in our research.

After a brief introduction of the interconnections between physical activity and planetary health, it is the objective of the symposium to present some projects that have begun to go beyond individual and public health and also consider issues of planetary health. This research will touch upon the issue of active transport, but also on the carbon footprint caused by sport and exercise. The symposium will give room for debate, to also allow raising some of the more difficult questions such as on how to deal with types of sport and exercise, that might be good for individual and public health, but have negative effects on the environment. Further objectives of the symposium are to create a better understanding on how the issue of planetary health might be integrated in physical activity research. Also, by bringing together researchers who are interested and have worked on this topic, to foster networking and collaboration.

